# Upcoming pharmacological developments in chronic hepatitis B: can we glimpse a cure on the horizon?

**DOI:** 10.1186/s12876-017-0726-2

**Published:** 2017-12-21

**Authors:** Sonia Alonso, Adriana-René Guerra, Lourdes Carreira, Juan-Ángel Ferrer, María-Luisa Gutiérrez, Conrado M. Fernandez-Rodriguez

**Affiliations:** Unit of Gastroenterology, Hospital Universitario Fundación Alcorcón, Av. Budapest-1, 28922 Alcorcon, Madrid Spain

**Keywords:** Hepatitis B virus clearance, Latest pharmacological developments, HBV cccDNA, HBV functional cure, HBV eradication

## Abstract

**Background:**

Hepatitis B virus (HBV) chronic infection affects up to 240 million people in the world and it is a common cause of cirrhosis and hepatocellular carcinoma (HCC). HBV covalently closed circular DNA (cccDNA) plays an essential role in HBV persistence and replication. Current pharmacological treatment with nucleos(t)ide analogues (NA) may suppress HBV replication with little or no impact on cccDNA, hence lifelong treatment is required in the vast majority of patients. Clearances of intrahepatic cccDNA and/or HBsAg are critical endpoints for future antiviral therapy in chronic HBV. Recent promising developments targeting different molecular HBV life cycle steps are being pre-clinically tested or have moved forward in early clinical trials.

**Methods:**

We review the current state of the art of these pharmacological developments, mainly focusing on efficacy and safety results, which are expected to lay the ground for future HBV eradication. An inclusive literature search on new treatments of HBV using the following electronic databases: Pubmed/MEDLINE, AMED, CINAHL and the Cochrane Central Register of Controlled Trials. Full-text manuscripts and abstracts published over the last 12 years, from 2005 to March 2011 were reviewed for relevance and reference lists were crosschecked for additional applicable studies regarding new HBV antiviral treatment.

**Results:**

HBV entry inhibitors, HBV core inhibitors, HBV cccDNA transcripts RNA interference, HBV cell apoptosis inducers, HBV RNA, viral proteins and DNA knock down agents, HBV release inhibitors, anti-sense nucleosides, exogenous interferon stimulation, interferon response stimulation and HBV therapeutic vaccines were reviewed.

**Conclusion:**

This review will provide readers with an updated vision of current and foreseeable therapeutic developments in chronic hepatitis B.

## Background

The percentage of the world’s population chronically infected with the Hepatitis B virus is approximately 5%. This infection is the main cause of chronic liver disease and hepatocellular carcinoma (HCC) globally. From 1990 to 2005 there has been a global decrease in HBV chronic infection prevalence due to expanded vaccination [1]. However, this condition is still a leading cause of global mortality and its overall burden and relative rank of mortality and disability rose between 1990 and 2013 [2]. Although there is important geographic variation with 75% of the infected population living in China and the highest prevalence occurring in central sub-Saharan Africa [1], HBV represents an important global public health issue with a considerable burden to almost all health systems [3–5]. Whilst universal vaccination might provide a key step forward in the HBV global eradication horizon, current therapies only confer clinical control through antiviral activity with few patients achieving HBsAg loss [6], a functional cure not equivalent to viral eradication. In the era of direct antiviral agents (DAAs), more than 95% of hepatitis C virus (HCV) patients achieve viral eradication, which contrasts with the therapeutic outcome in HBV chronic infection. HBV therapeutic guidelines recommend treatment to prevent liver disease progression, decompensation of cirrhosis and HCC development [7, 8]. The current standard of care includes administration of nucleos(t)ide analogues (NAs) and peginterferon. Cure of HBV infection is uncommon, influenced by the tenacity of covalently closed circular DNA (cccDNA) in the hepatocytes nuclei. Interferon-based therapies are usually recommended for 48 weeks and may provide more benefit in HBeAg positive patients with low viremia, elevated ALT and HBV genotype-A [9, 10] however, the benefit in terms of HBV clearance is still low. NAs (Entecavir and Tenofovir), inhibit the HBV polymerase activity and thus viral replication, but with no major impact on cccDNA which is used to transcribe viral RNAs. Consequently, NAs do not prevent the expression of HBV genes from cccDNA or the production of sub-viral particles. Long-term NAs administration achieves HBV eradication in only 5–8% of cases [6, 11]. Hence, the vast majority of patients require lifelong treatment and rebounding of viral replication frequently follows drug cessation. Furthermore, HCC risk is reduced but not eliminated, even after long-term effective viral suppression [12]. On the long-term, sustained virological response (SVR) or functional cure occurs in less than 10% of patients [13, 14]. Hence, new therapies to eliminate HBV are needed. Research developments in HBV molecular virology have resulted in relevant advances in discerning potential therapeutic targets. This review outlines recent pre-clinical and early clinical drug developments aimed at HBV clearance including HBV entry inhibitors, 2^nd^ generation Core inhibitors, TLR (*toll-like receptor*) agonists, anti-sense nucleotides and cccDNA targeting agents.

## HBV Life cycle

The viral entry to hepatocytes starts with a reversible attachment to the low affinity host cell surface heparan-sulfate proteoglycans. This is continued with a more specific attachment of the receptor-binding region of pre-S1 to the extracellular loops of the hepatocyte specific receptor sodium taurocholate co-transporting polypeptide (NTCP), a multiple transmembrane transporter [15]. NTCP discovery has been a significant breakthrough in the field of HBV molecular biology as it has allowed the development of reliable HBV cell cultures to explore both the HBV and HDV life cycle as well as in vitro drug testing. The binding of the pre-S1 region to NTCP elicits endocytosis before the HBV nucleocapsid transfers to the cell nucleus [16]. However, key steps in replication such as viral particle and cell membrane fusion, uncoating, and transference of HBV relaxed circular DNA (rcDNA) to the nucleus, are still incompletely understood. Once in the nucleus, rcDNA is transformed into covalently closed circular DNA (cccDNA), which acts as the template for the transcription of all viral mRNAs and pregenomic RNA (pgRNA). The pgRNA is encapsidated with the P protein. Once in the nucleocapsid, the pgRNA is reverse transcribed into negative-strand DNA. From the negative-strand DNA, the rcDNA is produced by plus-strand synthesis and the nucleocapsids are then either re-imported to the nucleus for cccDNA amplification or else enveloped and released via the endoplasmic reticulum (ER). Inhibition of these stages is an important target of drugs under development [17]. Epigenetic modifications such as histone acetylations and methylations and the HBx protein regulate the transcriptional activity of cccDNA [18].

**Fig. 1:**
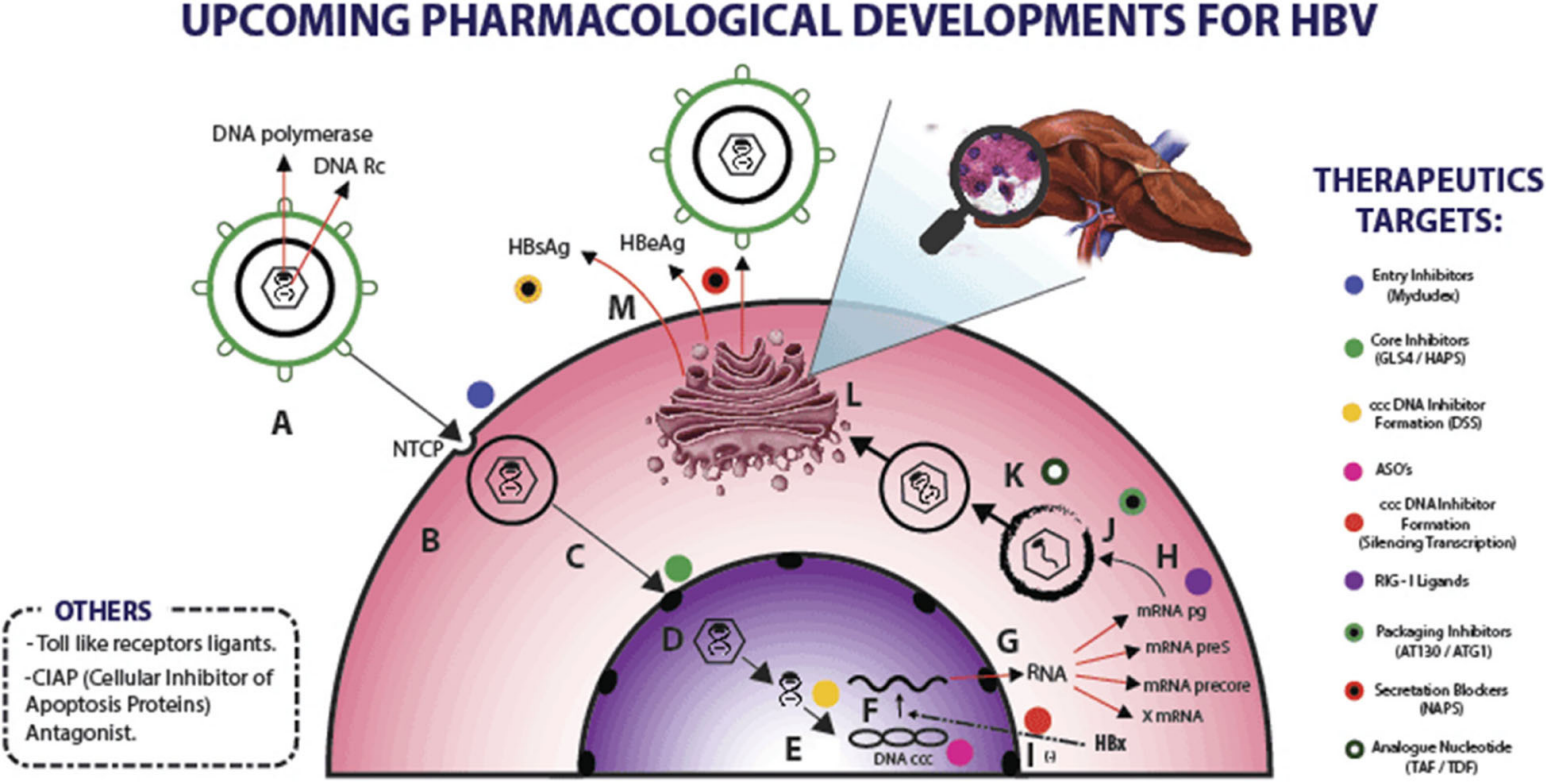
HBV Life cycle and therapeutic targets. The colour dots represent different drugs that work in many stages of the life cycle: **a** Binding HBV to NCTP receptor and endocytosis. **b** Uncoating nucleocapsid protein and release into cytosol. **c** Nuclear transport and bind to cell nucleus. **d** Shell of capsid disintegration and release of RNA. **e** rcDNA conversion to cccDNA. **f** Transcription. **g** RNA exportation from cell nucleus to cytosol. **h** Translation. **i** HBx stops transcription silence. **j** New nucleocapsids formed with RNA. **k** DNA synthesis from RNA (Target of Nucleos(t)ide analogues). **l** Nucleocapsid envelopment in Golgi apparatus. **m** HBsAg + HBeAg secretion and new viral particle

Furthermore, there are viral and host factors involved in the synthesis, stability and transcriptional regulation of cccDNA synthesis. One of them has recently been discovered as the tyrosyl-DNA phosphodiesterase 2 (TDP2) which is involved in the first step of cccDNA formation and offers a potential target for the development of drugs directed at HBV eradication [19]. In addition, an inactivation of cccDNA transcription by hyperchromatination, has been pointed to as another potential tool to achieve functional cure (“locking” cccDNA) (Fig. 1).

## Current therapies

Current standard of care includes peginterferon alfa2a, and NAs (tenofovir and entecavir) as first line therapies to suppress HBV viral replication, which is followed by a biochemical response and improvement in liver histology [20, 21].

Peginterferon α has antiviral, anti-proliferative, and immunomodulatory effects. In hepatitis B e antigen (HBeAg)-positive patients, 1-year peginterferon monotherapy leads to HBeAg and HBsAg seroconversion in 29–32% and 3–5% at 6 months of follow-up, respectively [9]. ALT normalization and sustained viral suppression of HBV (DNA < 400 copies/ml) is achieved in about 15% of HBeAg negative patients [10].

Importantly, peginterferon-α treatment allows finite treatment duration with a 4% of HBsAg loss reported at 6 months off-therapy, which progressively increased to 11% after 4 years of follow-up [9, 10]. However, its long-term use is limited by the side effects.

Although both entecavir (ETV) and tenofovir (TDF) are potent nucleos(t)ide HBV polymerase inhibitors with a high genetic barrier to drug resistance, they do not affect the transcriptional activity of cccDNA and life-long treatment to achieve a significant reduction of the cccDNA pool would be required. They also have a modest effect on the HBsAg level as well as poor immunological control [22]. Nevertheless, either HBe Ag-positive or negative treatment-*naïve* patients achieve more than 90% rate of HBV undetectability after long-term treatment with ETV [14] and TDF [13]. On the other hand, HBeAg seroconversion occurred in 21% of patients after 1-year of ETV and TDF therapy [14, 23], and more importantly, HBsAg loss was achieved in 11.8% of HBeAg-positive patients after 7 years of TDF treatment. 5-year cumulative probability of genotype resistance in patients treated with ETV was 1,2% [24] and resistance to TDF has not been reported after 7 years of treatment [13].

This maintained viral suppression is associated with improvement in necro-inflammation and fibrosis scores in most patients [20] and to a reduction in HCC risk in patients receiving ETV compared to untreated historical controls in an Asian [25] but not in a Caucasian population [26]. Although resistance rates are so far extremely low in the case of ETV and not yet described with TDF, concerns about long-term resistance and safety remain as critical unmet needs.

Long-term, perhaps indefinite, NA therapy is normally administered to HBeAg-negative patients. Recent evidence from a Greek study suggests that long-term (≥ 4-year) ETV/TDF therapy may be safely discontinued in noncirrhotic HBeAg negative patients, particularly with mild to moderate fibrosis, although retreatment rates were 0%, 15%, 18%, 24%, 26% at 1, 2, 3, 6, 9 months after ETV/TDF cessation [27].

Combination therapy with IFNα and NAs, add-on or switch may have a synergistic effect by combining antiviral and immunomodulatory mechanisms. Although TDF and peginterferon-alfa2a combination resulted in an increased rate of HBsAg loss than either therapy alone, this rate (9.1%) still remains low [23]. Whilst add-on ETV to peginterferon treatment in HBeAg positive patients failed to show significant benefit [28], switch to peginterferon in HBeAg positive patients on ETV achieved higher HBeAg seroconversion and 8.5% of HBsAg loss. Predictors of response included an early-on decline of HBsAg or baseline levels of < 1500 IU/ml [29]. Recently a multicentre randomised trial comparing add-on or switch to peginterferon alpha 2b for 48 weeks in HBeAg patients on NA therapy, compared to continuing NA, showed that HBeAg loss or decrease in HBsAg levels >1 log at week 72 was significantly higher in the add-on but not the switch arm, compared to the controls. This suggests that compared to the other two options, add-on therapy is a superior strategy [30]. A recent randomized controlled open trial evaluated the efficacy and safety of addition of a 48 week course of peginterferon in HBeAg-negative chronic hepatitis B patients on NA therapy with undetectable HBV DNA for a least 1 year. Addition of Peginterferon to NAs therapy in 92 patients was poorly tolerated with no differences in HBsAg clearance, when compared to 93 patients who continued NA therapy alone (difference 4,6% [95% CI -2·6 to 12·5]; *p* = 0·15) [31]. To date, combination treatment strategies are not recommended and require further assessment of efficacy and safety. Therefore, new therapies to eliminate intrahepatic cccDNA are prompted for patients with an increased risk of developing cirrhosis and HCC.

## New NAs

Tenofovir alafenamide (TAF) is a second-generation pro-drug of tenofovir and is mainly metabolized intracellularly to tenofovir diphosphate showing lower levels of tenofovir in plasma than TDF [32]. Recently two phase III studies, showed non-inferior efficacy of 25 mg of TAF to TDF on virological suppression at week 48 in HBeAg positive and negative patients. Patients who received TAF had less changes in bone and renal parameters due to the absence of renal organic anion transporters, OAT1 and OAT3-dependent cytotoxicity [33, 34].

Besifovir (LB80380) is a novel guanosine analogue with potent anti-HBV activity and works even against viruses resistant to approved NAs [35]. In a multicenter randomised trial, besifovir showed similar rates of virological response and HBeAg seroconversion compared to entecavir [36]. Although both TAF and besifovir might represent important advances, they do not clear intrahepatic cccDNA, and thus do not achieve a cure for HBV infection alone.

### Novel developments and potential therapeutic approaches

Extensive research on HBV life cycle and virus-host interactions has shed light on potential viral and host targets that are accountable for persistent HBV infection (Table 1).

**Table 1:** Novel agents against HBV and phase of development

Mode of action	Target	Drug	Clinical phase
Direct acting antivirals			
Polymerase inhibition	HBV polymerase	Tenofovir alafenamide	Phase 3
		Besifovir	Phase 3
Entry inhibition	NTCP	Myrcludex-B	Phase 2a
Core inhibitors	Nucleocapsid assembly	NVR 3–778	Phase 2
		AT-61, AT130, Bay 41–4109	Preclinical
Cleavage of DNA	ccc-DNA	ZFNs,TALENs,CRISPR/Cas	Preclinical
Inhibition of ccc-DNA formation		CCC-0975 and CCC-0346	Preclinical
Non-cytolitic cccDNA degradation by inducing APOBEC3A and APOBEC3B		Lymphotoxin-b receptor agonist	Preclinical
Apoptosis induction by inhibiting cIAPs	CIAPs	Birinapant	Phase 1
Knock down HBV RNA, viral proteins and HBV DNA	HBV RNA	ARC-520, ARC-521	Phase 2
Block release of HBsAg		REP-2139	Phase 2
Antisense nucleotides	Target RNA	ASOs	Preclinical
Host targeting agents			
Exogenous interferon stimulation	Innate immunity TLR7	Toll-like receptor (TLR) agonist (GS-9620)	Phase 2
Stimulate IFN response	Innate immunity RIG-I	SB 9200	Phase 2
Therapeutic vaccination	Adaptive immunity	GS-4774 (Tarmogen)	Phase 2b
		ABX203	Phase 2b

*APOBEC* apolipoprotein B mRNA editing enzyme, catalytic polypeptide 3A and 3B, *ASO* antisense nucleotides, *cccDNA* covalently closed circular DNA, *CIAPs* Cellular inhibitor of apoptosis proteins, *CRISPR/Cas* clustered regulatory interspaced short palindromic repeats (CRISPR) and CRISPR associated (Cas) systems, *NTCP* sodium taurocholate co-transporting polypeptide, *RIG-I* Retinoic acid-inducible gene, *TALENs* transcription activator-like effector nucleases, *ZFNs* zinc-finger nucleases

#### HBV attachment inhibitors

The basis of HBV entry inhibitors is the disruption of viral propagation that potentially could prevent post-exposure infection in some situations, such as after liver transplantation and in neonates of infected mothers. Moreover, addition of entry inhibitors to other antivirals could allow the inhibition of de novo infection of *naïve* hepatocytes and elimination of infected hepatocytes through induced immunomodulation while allowing the development of uninfected hepatocytes, thereby “clearing” the liver from HBV [37]. As previously commented, NTCP has been identified as a specific binding receptor of the pre-S1 domain of the HBV envelope protein for HBV entry into the host cell [15], therefore, is a potential therapeutic target.

Myrcludex-B, is a synthetic lipopeptide coming from the pre-S1 domain of the HBV envelope protein, which targets NTCP and inhibits HBV entry by competing for the NTCP receptor [37, 38]. This compound was well tolerated at the highest intravenous dose of 20 mg in 36 healthy volunteers [39]. In immunodeficient humanized mice (human liver chimeric uPA/SCID mice) infected with HBV, the serum viral load and HBsAg levels were reduced, showing its effect on the inhibition of amplification of intrahepatic cccDNA and preventing intrahepatic viral spreading [40]. In 2012, Phase I clinical trials in HBV patients were completed [38]. In a phase IIa clinical study, 75% of patients achieved > 1 log decrease in serum HBV DNA with once daily subcutaneous Myrcludex B. Higher doses were related to a clinically non-significant raise in serum bile acid levels [41]. Among patients with hepatitis D virus (HDV) coinfection, monotherapy with Myrcludex B showed a significant reduction on HDV RNA serum levels and ALT normalization. When added to peginterferon-alfa2a a synergistic antiviral effect on HDV RNA and HBV DNA was observed [42].. This pilot study was a sub-study of a phase Ib/IIa randomized, open-label clinical trial which compared daily myrcludex B vs. entecavir administration in patients with CHB. A liposomal formulation of Myrcludex B allows oral administration and long-term storage [43].

Combination regimens of Myrcludex B with immunomodulator or antiviral agents may improve efficacy and further clarify safety concerns. The combination of NAs and entry inhibitors might accelerate the elimination of infected cells preventing reformation of cccDNA after removal of NAs and induction of relapse and subsequently viral clearance.

Heparan Sulfate and Glypican 5 [44] are other NTCP co-receptors, but their role as HBV entry inhibitors has not yet been evaluated.

Polyethylenimines (PEI) are polymers that block the interaction between viruses and proteoglycans on the cell membrane, thus preventing viral entry. This polymer reduced the production of HBsAg and core-associated HBV DNA by 80% and more than 60%, respectively, when equated to the control in HepG2-hNTCP cells [45].

#### Core inhibitors

Pregenomic RNA encapsidation is vital for the subsequent HBV-DNA synthesis. Core inhibitors may additionally inhibit capsid disassembly at the nuclear pore and affect occupancy of cccDNA in the nucleus. The hetero-aryl-dihydropyrimidines (HAPs) are potent inhibitors of capsid assembly with the construction of aberrant core particles. BAY 41–4109 is one of these compounds that has been tested in different HBV models [46, 47] and achieved a rapid reduction in HVB DNA replication, but a rapid rebound after the end of the treatment in an animal model [48]. GLS4 is another member of the HAP family with in vitro *activity* inhibiting HBV replication of adefovir resistant strains [49] and entered early clinical development in China. GLS4JHS and Ritonavir have been shown as a safe combination and revealed a significant and rapid reduction in HBV-DNA and HBsAg levels in patients with chronic HBV infection [50]. Recently, third-generation 4-H HAPs have shown improved anti-HBV activity in vitro and in vivo and better drug-like properties compared to the first- and second-generations. They have been subsequently selected for further development as oral anti-HBV infection agents [51].

Both phenyl-propenamide AT-61 and AT-130 affect HBV-RNA packaging and formation of capsids [52], but must be proven in clinical trials.

Compounds with more advanced research are sulphamoyl-benzamide derivatives, which inhibit the encapsidation of viral pregenomic RNA into nucleocapsids and block the secretion of virions and particles containing RNA. A prototype of these core inhibitors, NVR 3–778, has shown superiority over peginterferon in the humanized uPA/SCID mouse model [53] and showed a good safety profile in a phase Ia trial in healthy adult volunteers [54]. Different doses of NVR 3–778 were well tolerated in a phase Ib clinical trial enrolling 36 HBeAg positive chronic hepatitis B patients. Significant HBV-DNA decline was observed only with the higher 1200 mg (600 mg b.i.d.) dose [55]. Currently, NVR 3–778 in combination with peginterferon, as well as with nucleoside analogs, is being explored. In a four week interim analysis, NVR 3–778 (400 and 600 mg) plus peginterferon was associated with reduction of both HBV DNA (1.97 log DNA-HBV for peginterferon and NVR 3–778 combination) and HBeAg, but not to HBsAg reduction, which is likely due to the short treatment duration [56, 57].

When AB-423, which is a novel antiviral agent, is combined with nucleoside or RNAi agents in vitro it has shown a potent inhibition of HBV replication.. Its high potential is sustained by inhibition of pgRNA encapsidation and the formation of cccDNA. Evaluation of AB-423 for advancement into clinical development is underway. [58].

Core Protein Assembly Modifiers (CPAMs) are compounds that target core protein. A recent study comparing a series of CPAMs with entecavir has shown their capacity in suppressing both HBV replication and formation of cccDNA. They can inhibit new rcDNA synthesis by interfering with pgRNA encapsidation and suppressing HBV DNA replication. Reductions in HBeAg, HBsAg and pgRNA levels in cell cultures prove its ability to block HBV de novo infection [59], unlike ETV.

#### cccDNA inhibitors

As commented, the cccDNA mini-chromosome is a key intermediate in the HBV life cycle, is responsible for HBV infection persistence and resides in the nucleus of infected cells (Fig. 1). There are several options for cccDNA targeting: inhibition of its formation, silencing its transcription or eliminating already existing cccDNA.

Another challenge is the lack of standardized assays for specific cccDNA quantification in cells and tissues, for the discrimination between rcDNA and cccDNA pools and markers of cccDNA activity to assess efficacy of treatments.

- Inhibition of cccDNA formation: di-substituted sulfonamide (DSS) termed CCC-0975 and CCC-0346 has proven its capacity to interfere with the conversion of rcDNA into cccDNA in cell culture [60]. As cccDNA has a long life, these compounds would have a role during the first phase of infection or high hepatocyte turnover [61].
- Silencing cccDNA transcription: cccDNA transcription and HBV gene expression are controlled by the regulation of HBV chromatin and cccDNA-bound histone post-translational modifications (PTMs) [62]. The ability of Peginterferon to inhibit cccDNA transcription relies on the reduction of cccDNA-bound histones acetylation [63]. HBx may represent a target for direct-acting antivirals as it appears necessary to block cellular factors that inhibit cccDNA transcription [64]. Some recent work has defined the role of the HBx protein interacting with “structural maintenance of chromosome” Smc complex Smc5/6, which inhibits extrachromosomal DNA transcription. HBx relieves the inhibition of HBV gene expression by destroying this Smc5/6 complex [65, 66]. Another study determined that the Smc5/6 complex limits hepatitis B virus transcription when confined to ND10 (Nuclear Domain 10) in human hepatocytes and that this association is important for transcriptional silencing of cccDNA in the absence of HBx [67]. Induction of PTMs on cccDNA bound histones by small compounds [68] can reduce cccDNA transcription and therefore inhibit viral replication, opening the possibility for an epigenetic silencing of cccDNA as a new approach. Although it must be confirmed *in vivo*, this strategy could achieve a functional cure.
- Elimination of cccDNA: Non-cytolytic elimination (‘curing’), or destruction of all cells harbouring cccDNA by T cells (‘killing’) and replacement by non-infected cells are the two ways for cccDNA clearance from hepatocytes [69]. Cytokines and downstream effectors play an important role, which is not yet completely known. In this way, the results of a recent study showed T-cells derived IFNγ and TNF-α to decrease levels of HBV cccDNA in hepatocytes by inducing deamination and subsequent cccDNA decay in vitro [70]. Lymphotoxin-b receptor agonists activate apolipoprotein B mRNA editing enzyme, catalytic polypeptide 3A and 3B (APOBEC3A and APOBEC3B) cytidine deaminases in HBV infected cells, inducing non-cytolytic cccDNA degradation [61]. Nevertheless, a fraction of cccDNA may persist refractory to immune-mediated clearance and degradation. New tools for targeting and cleaving cccDNA have been explored in cell models, such as zinc-finger nucleases (ZFNs), transcription activator-like endonucleases (TALENs) or the RNA-guided clustered regularly interspaced short palindromic repeats (CRISPR)/Cas system. ZFNs target sequences within the HBV polymerase, core and X genes, and break the DNA double strand with imprecise repair that leads to mutation which inactivates HBV genes. In a recent study [71], delivery of 3 HBV specific ZFNs, using self-complementary adeno-associated virus vectors, achieved total inhibition of HBV DNA replication and manufacture of infectious HBV virions in HepAD38 cells. In vivo murine hydrodynamic injection model of HBV replication with TALEN led to a targeted mutation in approximately 35% of cccDNA molecules without evidence of toxicity [72]. Finally, a recent study showed that over 90% of HBV DNA was cleaved in by Cas9 [73]. Despite their potential, it is necessary to elucidate efficacy of these compounds in animal models of chronic HBV infection prior to clinical development and to assess aspects related with off-target effects affecting the host genome.

#### Apoptosis inductors: SMAC mimetic drugs

Cellular inhibitor of apoptosis proteins (cIAPs) prevent TNF-mediated killing/death of infected cells, thus impairingthe clearance of HBV infection [74]. Drug inhibitors of cIAPs are also known as Smac (second mitocondria-derived activator of caspase) mimetics, because they mimic the action of the endogenous protein Smac/Diablo that antagonizes cIAP function. Recent studies have shown that birinapant and other Smac mimetics produced a rapid decrease in serum HBV-DNA and HBV surface antigen and promoted the removal of hepatocytes containing HBV core antigen in an immunocompetent mouse model of chronic HBV infection. Liver enzymes were transiently elevated showing non-significant liver damage related to the action of birinapant. The effect of birinapant and ETV in combination was higher than either drug alone in promoting clearance of serum HBV DNA with no overt evidence of toxicity [75]. In 2015, a phase II study of birinapant for the treatment of HBV was initiated.

#### Inhibition of HBV gene expression

As collapse and dysfunction of HBV-specific T-cell immunity in chronic hepatitis B might be related to the presence of increased levels of viral load, a reduction or disruption of HBV gene expression may be a potential tool to achieve immune restoration [62].

- *Secretion pathways*: Nucleic Acid Polymers (NAPs), blocked the release of HBsAg. REP9-AC (REP 2055), a 40-nucleotide DNA polymer, led to rapid clearance of serum HBsAg and anti-HBs appearance [76]. REP-2139, a modified compound with no inflammatory effect showed a synergistic antiviral effect when peginterferon was added-on after HBsAg clearance and also in combination with peginterferon in patients with HBeAg positive chronic HBV infection [77].. In both studies NAP monotherapy for 40 weeks, resulted in 2–7 log reductions of serum HBsAg, 3–9 log reductions in serum HBV DNA and development of serum anti-HBsAg antibodies. In the randomized, controlled trial REP 401 protocol (NCT02565719), triple antiviral therapy with NAPs, peginterferon and TDF in Caucasian patients with HBeAg negative chronic HBV are currently being evaluated. Efficacy and tolerability of REP 2139 and REP 2165 in combination with peg-IFN and TDF have been proven in 34 patients with HBeAg negative chronic HBV infection. 9/9 patients receiving REP 2139 and 7/9 patients receiving REP 2165 achieved >1 log reduction in serum HBsAg [78]. Larger controlled studies are needed to confirm whether immune restoration occurs after NAPs induced HBsAg clearance.
- *RNA interference:* In gene expression disruption RNA interference is one of the most widely used approaches. As mentioned above, high levels of viral antigens such as HBsAg might alter HBV-specific T-cell immunity in chronic hepatitis B. Thus, it is conceivable that reduction of HBV gene expression might lead to immune restoration. ARC-520 is a combination of a hepatocyte-targeted, N-acetyl-galactosamine conjugated Melittin-like peptide with a liver-tropic cholesterol-conjugated small interfering RNA (siRNA). It is directed against conserved HBV RNA sequences that require intravenous administration to effectively knock down HBV-RNA, HBsAg and DNA levels in chimpanzees. Of note, ALT flares were observed reflecting immune reconstitution [79, 80]. Data from a phase IIa clinical trial confirm tolerability and efficacy of ARC-520 showing significant, dose-dependent reduction in HBsAg for up to 57 days in CHB patients [81, 82]. The beneficial effect of multi-dose treatment with ARC-520 in chimps previously treated with NAs may lead to the effective knockdown of target genes with no development of drug resistance by triggering two sites [83]. Furthermore, a recent study has shown that ARC-520 (siRNA) and entecavir led to quick HBV-DNA suppression in all HBeAg positive patients achieving up to 5.5 log reductions of HBV-DNA. This also occurred in all HBeAg negative treatment naïve patients achieving decreases up to below the limit of quantitation. After a single dose was administered to HBV patients, ARC-520 inhibited HBV cccDNA-derived mRNA, as up to a 2-log viral protein reduction was observed [84].
  A preclinical study, using cell culture models, found that in HepBHAe82 cells, the capsid inhibitor AB-423 in combination with a second-generation siRNA agent, ARB-1740, displayed synergistic activity against HBV relaxed circular DNA. Their activity also led to an important decline in HBV DNA, whilst maintaining the serum HBsAg inhibition mediated by ARB-1740 and the HBeAg level when added to Peginterferon or entecavir during a 28 day period [85].
  ARB-1467 contains three double-stranded siRNAs, which target three different sites in the viral genome to realize post-transcriptional gene suppression of HBV proteins. These proteins are generated from both cccDNA and integrated DNA, including the surface antigen (HBsAg). The safety and efficacy of ARB-1467 was evaluated in a recent study over a period of 12 weeks in 24 subjects and showed declines in HBsAg levels with single and multiple doses [86].
  BB-103 is a recombinant AAV8 vector which is designed to treat chronic HBV infection using RNAi, and targets three sequences in the Core, S-antigen and X protein regions on the HBV viral RNA. When combining a single dose of this compound with Peginterferon or entecavir in a mouse model, HBV DNA was reduced nearly 4 log while also achieving a 2 log drop in HBsAg [87].
  Recently, a biodegradable nanoparticle, which can deliver HBV-targeting unlocked nucleomonomer agent (UNA) oligomers successfully to hepatocytes, has been proven to show an excellent tolerability. A combination of three UNA oligomers with capacity to target all viral transcripts and cover all HBV genotypes has shown potent activity against HBV in HBV-infected human hepatocytes and in two mouse models of HBV infection [88].

#### Cyclophilin inhibitors

Cyclophilins are cytoplasmic proteins used by several viruses for replication. Alisporivir, which was developed for the treatment of HCV, is a cyclophilin A inhibitor that has recently been shown to have an effect in reducing the replication of HBV DNA and HBsAg production and secretion and these effects were potentiated with telbivudine addition in a preclinical study [89].

#### Inmunological approaches

- Innate immune ligands

-*Toll-like Receptor (TLR) ligands:* The toll-like receptor family is an important regulator of innate and adaptive immune response through the recognition of foreign pathogens, which triggers the expression of genes involved in cytokines and antigen specific adaptive immunity. In mice models, HBV can be suppressed by TLR induced antiviral activity [90]. A TLR subfamily composed of TLR3, 7/8 and TLR9 recognize endosomal viral nucleic acids and induce a type-1 interferon response.

Short-term oral administration of the TLR7 agonist GS-9620 in chimpanzees, achieved reduction in serum and liver HBV-DNA and in HBsAg and HBeAg [91].GS-9620 stimulated the creation of interferon-alpha and other cytokines and chemokines, and activated interferon-stimulated genes and natural killer cells. A short course of oral GS-9620 in a phase Ib clinical trial did not show changes in HBsAg or HBV DNA levels although it was proven to be safe and well tolerated [92]. It is unknown whether longer therapy duration will improve this response. Results from a phase II study comparing GS-9620 during 4, 8 and 12 weeks in patients with viral suppression receiving tenofovir, did not show a decline of HBsAg levels [93]. As part of the SG-US-283-1059 study, 28 HBeAg negative with genotype D HBV infected patients in treatment with NUCs were randomized to receive either placebo or one of three different GS-9620 doses (1,2 and 4 mg, weekly for 12 weeks). All in vitro analyzed HBV-specific T cell responses were significantly stronger in virally-suppressed patients at baseline, mainly IFN-γ production and CD4 responses. When adding GS-9620 to NUC, production of IL-2 and CD8 responses are enhanced, as with the overall NK cell function, but changes in T cell/NK cell function and HBsAg decline were not correlated. The role of GS-9620 associated to other anti-HBV therapy might be investigated in different clinical settings [94].

-*STING agonists:* 5,6-dimethylxanthenone-4-acetic acid (DMXAA) is an agonist of the mouse stimulator of interferon genes (STING), and has been found to suppress HBV replication in mouse hepatocytes by inducing a robust cytokine response in macrophages followed by reducing the amount of cytoplasmic viral nucleocapsids. The STING agonist induced a cytokine response mainly by type I interferons, which is unlike the TLR agonists that induced a predominant inflammatory cytokine/chemokine response. [95].

-*RIG-I ligands:* Retinoic acid-inducible gene (RIG-I)-like RNA helicases (RLHs) recognize RNA in the cytoplasm and induce an IFN response as well as interfere with the interaction of the HBV polymerase with pgRNA to suppress viral replication. The active SB-9000, an oral dinucleotide prodrug, isomer products bind to RIG-I and NOD2 to stimulate an interferon response. Pretreatment with SB 9200 to induce a host immune response followed by ETV in woodchucks was found to have a significant reduction in viral DNA, RNA, and antigens compared to viral reduction with ETV followed by immune modulation. A Phase II clinical trial of SB 9200 alone and in combination with a nucleoside to treat chronic HBV is therefore planned [96].

b) Therapeutic vaccines

The basis for therapeutic vaccination is the achievement of breaking T cell tolerance to HBV proteins (HBsAg, HBcAg) and stimulation of HBV-specific T cell immunity in patients with chronic HBV infection. GS-4774 elicits an HBV-specific T-cell response through a heat-inactivated yeast-based therapeutic T-cell vaccine expressing a recombinant protein containing HBV core, surface, and X proteins. Recently, data from a 2phase IIb clinical trial have been published. 178 patients with non-cirrhotic chronic HBV infection with viral suppression by NUC therapy were randomized to continue antiviral therapy alone or receive NUC plus GS-4774 subcutaneously every 4 weeks. There were no significant differences between groups in mean HBsAg decreases from baseline to week 24 or 48 and no patient experienced loss of serum HBsAg [97]. The vaccine was safe and well tolerated in spite of poor clinical benefit. The investigators speculate with the possibility of better results in patients with shorter duration of infection.

Recent results from a randomized phase II study assessing the GS-4774 vaccine + TDF in 195 patients with chronic HBV who were not on antivirals evidenced modest reductions in HBsAg in the GS-4774 + TDF group when compared to the TDF group alone through week 48 and no patients lost HBsAg [93, 98].

A phase IIb study is currently evaluating the efficacy of a therapeutic vaccine composed of HBsAg and HBcAg recombinant proteins (ABX203) in HBeAg-negative patients after cessation of NA therapy [62].

In a recent preclinical mice model, reducing viral antigens with an adeno-associated virus targeting HBV transcripts via RNAi, prior to vaccination with a protein prime/modified vaccinia virus Ankara (MVA), induced higher HBs and HBc specific CD8 T-cell responses. This is due to the high levels of viral antigens promoting HBV tolerance [99].

Transfer of T-cells engineered to express a HBV specific T-cell receptor may reconstitute the immune response against HBV and reduced serological and intrahepatic viral loads in human liver chimeric mice [100].

#### Anti-sense nucleotides (ASOs)

ASOs are small single-stranded nucleic acids (8–50 nucleotides) which are complementary to their target RNA, and bind via base pairing, leading to the degradation of the specific target RNA. This is followed by reduction of HBV antigenemia with limited off-target effects [101]. Recently, in vitro and in vivo antiviral effects of first generation ASOs against HBV have been described [102, 103], but toxicity has limited their use in vivo*.* Second generation ASOs have provided improved potency, stability, specificity and safety [102]. A lead ASO identified in vitro, efficiently reduced HBV gene expression, replication, viremia and antigenemia in HBV transgenic mice. HBsAg decreased 2-logs in a week after a single ASO injection, as well as combined with ETV, while the NUC alone did not. Also, cccDNA-driven HBV gene expression is ASO sensitive in HBV infected cells *in vitro* [104]. Advantages of ASOs compared to siRNA compounds, such as ARC-520 [84], may consist in specific formulation required for the siRNA and the need of intravenous administration due to instability and delivery limitations of siRNA compounds.

#### Ribonuclease H inhibitors

The HBV ribonuclease H (RNaseH) has been recently evaluated as a drug target as it is essential for viral replication, since HBV is a DNA virus that replicates by reverse transcription via an RNA intermediate. N-hydroxyisoquinolinedione (HID), 3-hydroxypyrimidine-2,4-diones and α-hydroxytropolone compounds have confirmed its activity against viral replication by inhibiting RNaseH [105].

## Conclusions

**Fig. 2:**
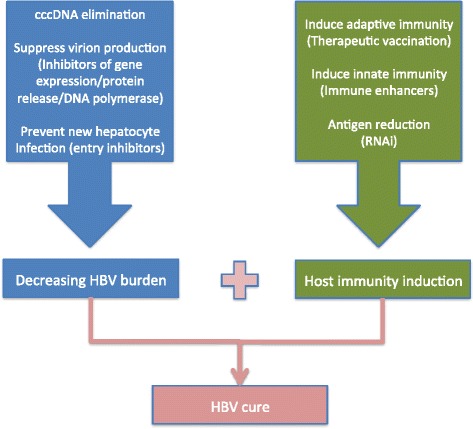
Suggested combination strategies to achieve HBV cure

Recent developments of new highly effective antiviral therapy against HCV infection have fueled the research for a cure of chronic HBV infection or HBsAg loss. Intranuclear cccDNA and HBV-DNA integration remain as critical barriers for HBV cure. Albeit, new preclinical and early-clinical development show promising proof of concept results, although most of the trials, even the more advanced, ones did not set HBsAg loss as a principal endpoint. Furthermore, which biomarkers are necessary to accurately assess sterilizing cure remains unclear. Thus, we are currently quite far from foreseeing which drugs or which combinations will eventually succeed in eliminating cccDNA. It is likely that combining agents directed at different specific steps in the viral life-cycle, including cccDNA targets, with those aimed at activating and restoring host anti-viral immunity will be needed to overcome HBV chronic infection. These therapeutic strategies are expected to be tested and enter clinical assessment in the next few years (Fig. 2). Additional concerns about safety of host targeting drugs remain to be elucidated. In this sense, a non-cytolytic purging of cccDNA containing hepatocytes stands out as the preferred approach.

Although we still seem to be a long way from definitely curing HBV infection, combining efforts of basic, translational, clinical research and awareness of regulatory agencies of the need for early combination trials, will hopefully pave the way to the cure of HBV infection in the next two decades.

## Abbreviations

ALT: Alanine Aminotransferase
cccDNA: Covalently circular closed DNA
cccDNA: HBV covalently closed circular DNA
DAAs: Direct Antiviral Agents
DMXAA: 5,6-dimethylxanthenone-4-acetic acid
ER: Endoplasmic reticulum
ETV: Entecavir
HBeAg: Hepatitis B e Antigen
HBsAg: Hepatitis B surface Antigen
HBV: Hepatitis B virus
HCC: Hepatocellular carcinoma
HCV: Hepatitis C virus
NAs: Nucleos(t)ide analogues (NAs)
NTCP: Sodium taurocholate co-transporting polypeptide
OAT: Organic anion transporters
pgRNA: Pregenomic RNA
rcDNA: Relaxed circular DNA
SMAC: second mitochondrial-derived activator of caspases
STING: stimulator of interferon genes
SVR: Sustained virological response
TAF: Tenofovir alafenamide
TDF: Tenofovir fumarate
TDP2: Tyrosyl-DNA phosphodiesterase 2
TLR: Toll-like receptor
UNA: Unlocked nucleomonomer agent
